# Ashwagandha as an Adaptogenic Herb: A Comprehensive Review of Immunological and Neurological Effects

**DOI:** 10.7759/cureus.96183

**Published:** 2025-11-05

**Authors:** Pallavi Prabhakar Jamnekar, Tejaswini Jeevan Dehankar, Rupali V. Bedre, Binthuja G Dharan, Bharat Agravat, Harsha Agravat

**Affiliations:** 1 Department of Pharmacology, Bhausaheb Mulak Ayurved Mahavidyalaya, Nagpur, IND; 2 Department of Physiology, Bhausaheb Mulak Ayurved Mahavidyalaya, Nagpur, IND; 3 Department of Siddha Yoga Medicine, Peripheral Pharmacovigilance Center, Thoothukudi, IND; 4 Department of Cosmetic and Dental Implants, Dr Agravat Wellness Center, Ahmedabad, IND; 5 Department of Ayurveda, Dr Agravat Wellness Center, Ahmedabad, IND

**Keywords:** adaptogen, ashwagandha, clinical trials, neuroimmune modulation, withanolides

## Abstract

Ashwagandha (*Withania somnifera*), a well-established herb in Ayurvedic medicine, is increasingly researched for its adaptogenic properties and regulatory roles in neuroimmune processes. The present review aims to integrate mechanistic, preclinical, and clinical evidence on ashwagandha’s immunomodulatory, neuroprotective, psychiatric, sleep-regulating, and anti-inflammatory activities, with emphasis on its bioactive compounds, such as withanolides, sitoindosides, and alkaloids. Such compounds modulate the hypothalamic-pituitary-adrenal (HPA) axis, inhibit NF-κB, induce Nrf2 activation, and affect gamma-aminobutyric acid (GABA)ergic signaling, collectively contributing to its anti-inflammatory, antioxidant, and anxiolytic actions. Clinical trials with standardized ashwagandha extracts have shown reductions in stress-related biomarkers, along with improvements in cognitive performance, sleep quality, and mood parameters. Neuroprotective actions have also been demonstrated in preclinical animal and cell models of Alzheimer’s and Parkinson’s disease (PD). The review does emphasize methodological shortcomings, however, such as heterogeneity in the preparation of extracts, small sample sizes, variability in endpoints, and possible funding-related biases. To further its clinical utility, subsequent studies should emphasize pharmacogenomic responsiveness given potential variability in cytochrome P450 (CYP)-mediated metabolism and genetic differences in stress-response pathways, along with standardized extract formulations and long-term, multicenter randomized trials with biomarker monitoring. By doing so, ashwagandha promises to be a science-validated botanical that can bridge conventional medicine with precision-guided, evidence-based therapeutic protocols in contemporary medicine.

## Introduction and background

Adaptogens are natural substances that help the body respond to various types of stress, thereby maintaining balance within the body [1]. More attention is being given to these substances in both integrative and conventional medicine because they can influence multiple signaling pathways with relatively few reported adverse effects [2].

In the past, adaptogens were a key part of Ayurveda and Traditional Chinese Medicine (TCM), where they were used to boost endurance, restore energy, and help people age more slowly [3]. Among the adaptogens, ashwagandha (*Withania somnifera*) is recognized for its broad effects on both the nervous and immune systems.

In India, North Africa, and the Middle East, the Solanaceae family includes the perennial shrub known as ashwagandha [4]. In Ayurveda, it belongs to the "*rasayana*" group, which are herbs thought to increase longevity, mental sharpness, and balance in the body [5]. Modern studies have found that ashwagandha contains more than 40 withanolides, sitoindosides, alkaloids, and flavonoids [6]. Withaferin A and withanolide D are the primary compounds responsible for the herb's adaptogenic, neuroprotective, and immunomodulatory properties [7]. They are known to affect important biological pathways such as the hypothalamic-pituitary-adrenal (HPA) axis, gamma-aminobutyric acid (GABA) signaling, and networks of pro-inflammatory cytokines [8].

**Figure 1:**
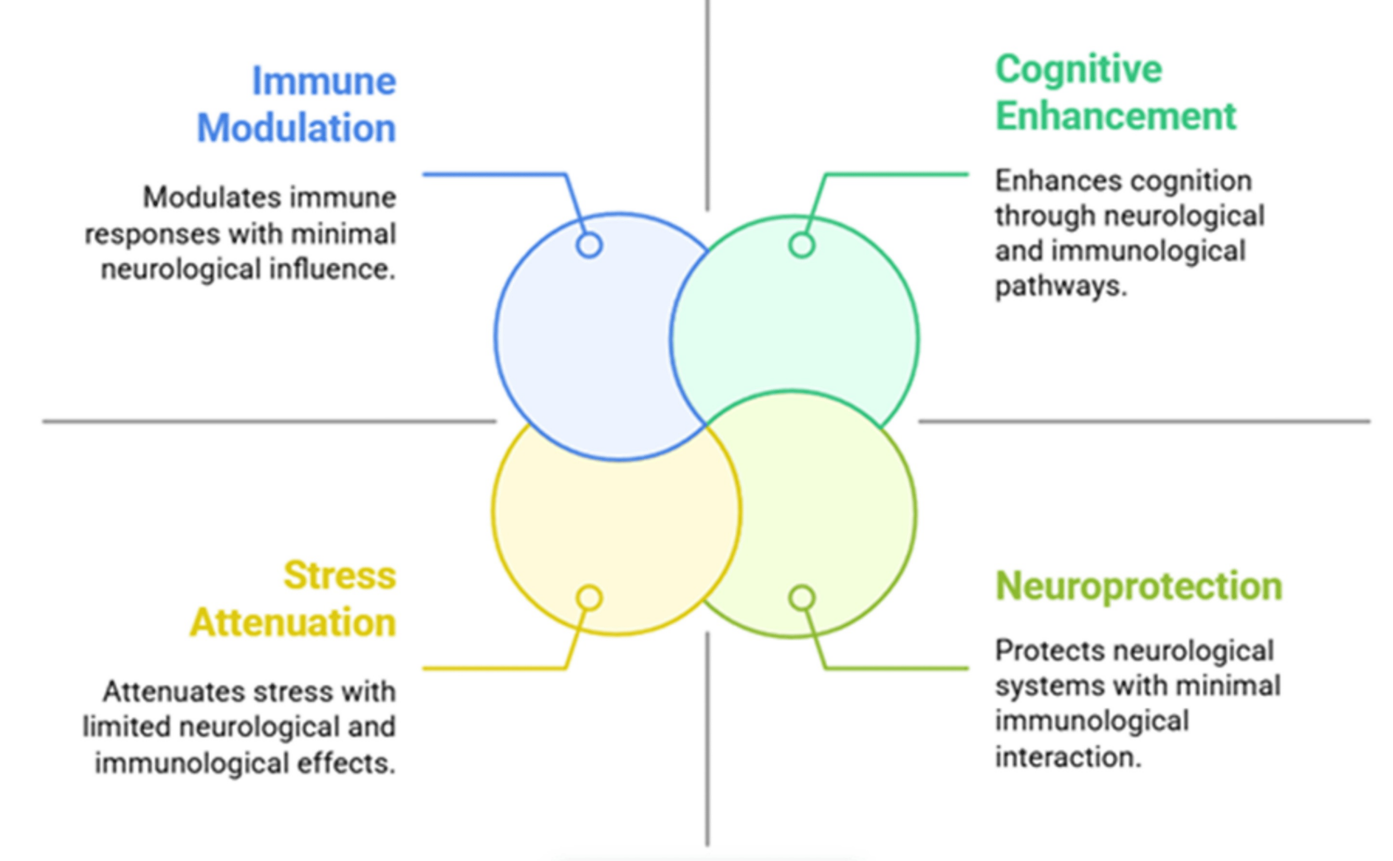
Core therapeutic domains of ashwagandha across neuroimmune function Created by authors using Napkin AI software (Napkin AI, CA, USA)

Since stress-related, immune-mediated, and neurodegenerative diseases are becoming more common, it has become necessary to find therapies that can address several aspects of these diseases [9]. Although many studies have been done on ashwagandha to see if it can ease anxiety, boost memory, change immune responses, and lower inflammation, the results are not fully consistent. The differences in how studies are done, how extracts are standardized, and the populations involved explain the inconsistent findings in the literature. Also, although studies often look at ashwagandha’s effects on the brain or immune system alone, there is not much research that explores how these two systems interact in ashwagandha’s adaptogenic role [10]. Figure 1 illustrates the multifaceted effects of ashwagandha, demonstrating its systemic adaptogenic potential through four primary domains: immunomodulation, stress attenuation, neuroprotection, and cognitive enhancement. These domains are interconnected via neuroimmune interactions, offering a framework for a system-based approach to evaluating ashwagandha’s adaptogenic efficacy.

Objectives of the review

This review seeks to consolidate mechanistic, preclinical, and clinical evidence regarding the adaptogenic potential of ashwagandha. Particular emphasis is placed on its immunomodulatory, neuroprotective, psychiatric, sleep-enhancing, and anti-inflammatory activities. The discussion explores how major bioactive constituents influence cytokine regulation, modulate the HPA axis, support cognitive and mood functions, and contribute to the regulation of sleep and circadian processes. Consideration is also given to the variability in extract preparations, the heterogeneity of study methodologies, and the overall quality of published findings. By integrating these perspectives, the review aims to present an updated framework for evaluating ashwagandha’s therapeutic relevance while underscoring the need for future research employing standardized, rigorously designed clinical trials.

## Review

Methods

Search Strategy

A systematic search of PubMed, Scopus, and the Cochrane Library was undertaken to identify relevant studies published between January 2015 and August 2025, with the final search conducted on August 2, 2025. The search strategy combined terms using Boolean operators: (“Ashwagandha” OR “Withania somnifera”) AND (“adaptogen” OR “immune modulation” OR “immunomodulation” OR “cognition” OR “memory” OR “clinical trial” OR “neuroprotection” OR “anti-inflammatory” OR “sleep” OR “anxiety” OR “depression”). Filters were applied to restrict results to English-language publications involving human participants or translational animal research.

Eligibility Criteria

Studies were included if they were peer-reviewed and reported original preclinical, clinical, or translational findings with quantitative outcomes related to neurological, immunological, psychiatric, sleep, or anti-inflammatory measures. Examples of eligible endpoints included cytokine profiles, stress biomarkers, standardized anxiety and depression scales, cognitive or memory tests, sleep efficiency metrics, and quality-of-life scores. Excluded were non-peer-reviewed material, case reports, reviews without original data, conference abstracts, and animal-only studies without translational relevance to human health.

Selection Process

**Figure 2:**
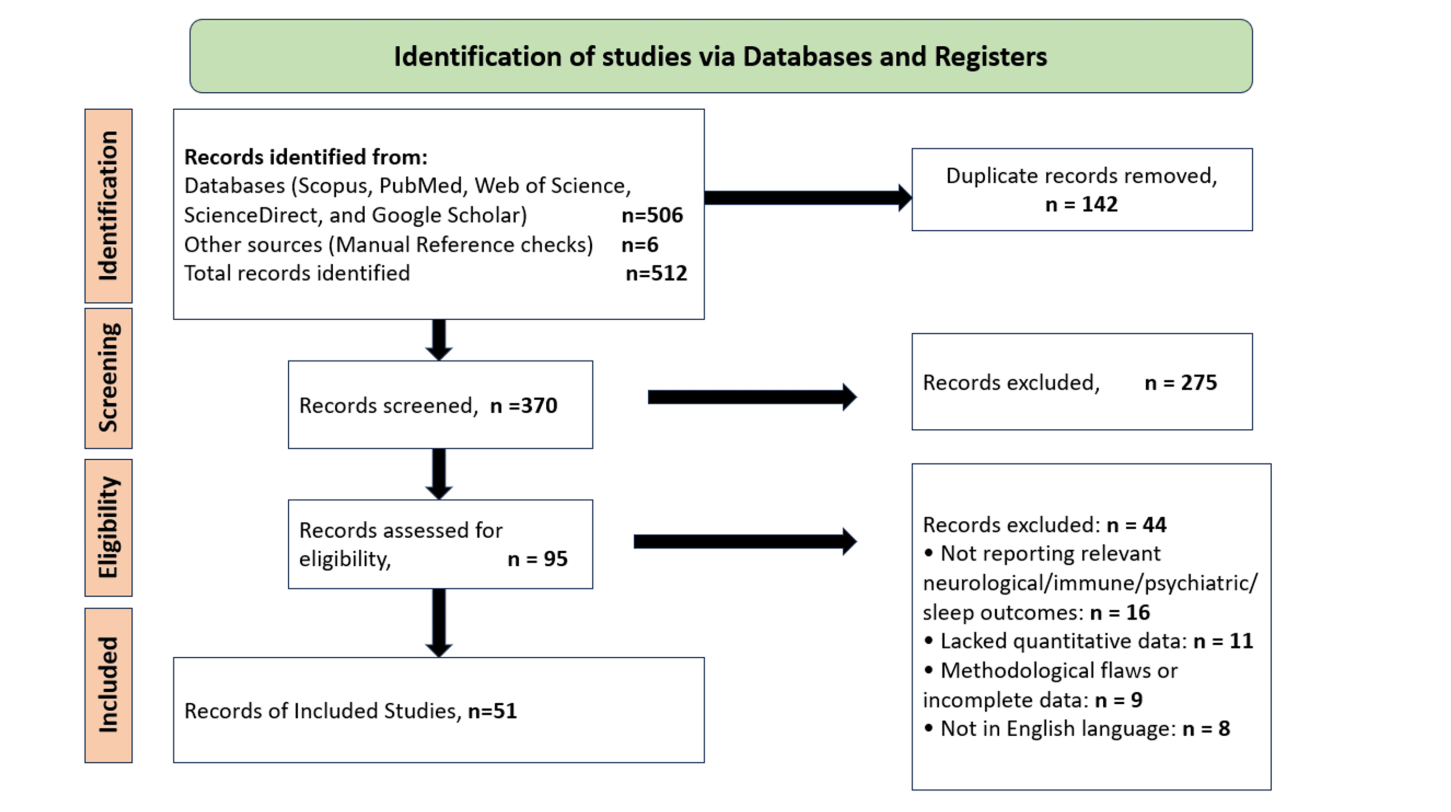
Preferred Reporting Items for Systematic Reviews and Meta-Analyses (PRISMA) flow diagram of study selection Created by authors using Microsoft PowerPoint (Microsoft Corporation, Redmond, Washington, United States)

Two reviewers independently screened titles and abstracts, followed by full-text evaluation of potentially eligible articles. Discrepancies were resolved through discussion until consensus was reached. Of the 95 full-text articles assessed for eligibility, 44 were excluded: 16 did not report relevant neurological, immunological, psychiatric, or sleep-related outcomes; 11 lacked quantitative data; nine had methodological flaws or incomplete reporting; and eight were published in languages other than English. The overall selection process is summarized in a Preferred Reporting Items for Systematic Reviews and Meta-Analyses (PRISMA)-style flow diagram (Figure 2), which illustrates the number of records identified, duplicates removed, studies screened, excluded, and finally included (n=51).

Assessment of Bias and Study Quality

Most clinical studies were of low to moderate quality, with recurring limitations including small sample sizes, short follow-up durations, and incomplete reporting of allocation concealment and blinding procedures. The risk of bias and quality assessment of the included studies are presented in Table 1.

**Table 1:** Risk of bias and quality assessment of included studies

Reference	Author, Year	Study Type	Tool Used	Overall Risk / Quality Judgment
[1]	Haber M, 2024	Review	N/A	Not applicable
[2]	Wróbel-Biedrawa D, 2024	Review	N/A	Not applicable
[3]	Mikulska P, 2023	Review	N/A	Not applicable
[4]	Guo S, 2024	Review	N/A	Not applicable
[5]	Al-Eisa RA, 2025	Review	N/A	Not applicable
[6]	Sobota W, 2024	Review	N/A	Not applicable
[7]	Kuśmierska M, 2024	Review	N/A	Not applicable
[8]	Speers AB, 2021	Review	N/A	Not applicable
[9]	Grabowski WK, 2024	Review	N/A	Not applicable
[10]	Hassan A, 2023	Review	N/A	Not applicable
[11]	Wiciński M, 2023	Review	N/A	Not applicable
[12]	D’Cruz M, 2022	Review	N/A	Not applicable
[13]	Tharakan A, 2021	RCT	RoB 2	Low risk
[14]	Wiciński M, 2024	Review	N/A	Not applicable
[15]	Wijeweera G, 2023	Review	N/A	Not applicable
[16]	KrishnaRaju AV, 2023	Preclinical	N/A	Not applicable
[17]	Khalid MU, 2025	Review	N/A	Not applicable
[18]	Sharma S, 2023	Review	N/A	Not applicable
[19]	Brekhman, 1969	Narrative review/experimental overview	N/A	Not applicable
[20]	Kumar U, 2024	Review	N/A	Not applicable
[21]	Lopresti AL, 2019	RCT	RoB 2	Low–moderate risk
[22]	Choudhary D, 2017	RCT	RoB 2	Moderate–high risk
[23]	Er B, 2025	Preclinical	N/A	Not applicable
[24]	Gopukumar K, 2021	RCT	RoB 2	Low–moderate risk
[25]	Kumar R, 2018	Observational	NOS	Moderate quality
[26]	Deshpande A, 2020	RCT	RoB 2	Low risk
[27]	Lopresti AL, 2019	RCT	RoB 2	Moderate risk
[28]	Choudhary D, 2017	RCT	RoB 2	Moderate risk
[29]	Manchanda S, 2017	Preclinical	N/A	Not applicable
[30]	Pandit S, 2024	RCT	RoB 2	Low–moderate risk
[31]	Salve J, 2019	RCT	RoB 2	Moderate risk
[32]	Verma N, 2024	RCT	RoB 2	Low risk
[33]	Chandrasekhar K, 2012	RCT	RoB 2	Low–moderate risk
[34]	Smith SJ, 2023	RCT	RoB 2	Low risk
[35]	Tiwari S, 2021	RCT	RoB 2	Low risk
[36]	Sukumar BS, 2021	Human trial	NOS	Moderate quality
[37]	Amir M, 2023	Review	N/A	Not applicable
[38]	Ossowska W, 2025	Review	N/A	Not applicable
[39]	Güllü H, 2024	Review	N/A	Not applicable
[40]	Dipankar SP, 2025	Review	N/A	Not applicable
[41]	Arshad MT, 2025	Review	N/A	Not applicable
[42]	Malec K, 2024	Review	N/A	Not applicable
[43]	Gupta S, 2021	Review	N/A	Not applicable
[44]	Xing D, 2022	RCT	RoB 2	Low–moderate risk
[45]	Iskandar AU, 2023	Review	N/A	Not applicable
[46]	Leonard M, 2024	RCT	RoB 2	Low risk
[47]	Alanazi HH, 2023	Review	N/A	Not applicable
[48]	Gupta V, 2024	Preclinical	N/A	Not applicable
[49]	Murthy MN, 2024	Preclinical	N/A	Not applicable
[50]	Syed AA, 2021	Review	N/A	Not applicable
[51]	Langade D, 2021	RCT	RoB 2	Low risk

Methodological quality was assessed at the study level. Randomized controlled trials were evaluated using the Cochrane Risk of Bias 2 (RoB2) tool, while observational studies were appraised with the Newcastle-Ottawa Scale [52].

Phytochemistry

Ashwagandha contains a diverse array of bioactive compounds that underpin its adaptogenic, neuroprotective, and immunomodulatory properties. The primary active constituents are withanolides, which share structural similarity with ginsenosides in *Panax ginseng*; however, this resemblance does not necessarily imply identical pharmacological activity, and comparative studies suggest that while both classes influence stress response and immune modulation, their specific molecular targets and efficacy profiles may differ [11]. Researchers have identified more than 40 withanolides, with withaferin A, withanolide A, and withanolide D most frequently studied for their ability to reduce inflammation, regulate apoptosis, and limit oxidative stress [12,13]. The glycowithanolides sitoindosides VII-X also belong to this group and demonstrate stress- and anxiety-reducing effects in animal models [14]. In addition, ashwagandha contains alkaloids such as somniferine, withanine, and tropine, which exert sedative, anticonvulsant, and hypotensive actions [15]. Flavonoids, coumarins, and amino acids contribute synergistically to its health benefits [16]. The method of extraction and preparation strongly influences both yield and bioactivity. Standardized extracts such as KSM-66 and Sensoril typically contain 1.5-5% total withanolides by weight [17]. However, many withanolides have low water solubility and undergo first-pass metabolism, which limits their absorption. To address this, researchers have developed lipid-based carriers, phytosomes, and nanoparticles. Recent advances in nanoformulation enable withaferin A to persist in plasma for longer and penetrate tissues more effectively, resulting in more consistent physiological effects [18]. In summary, ashwagandha’s complex chemical composition, particularly its withanolides, alkaloids, and polyphenols, forms the scientific basis for its classification as an adaptogen (Table 2). This phytochemical diversity enables a wide range of pharmacological actions, which are explored in subsequent sections.

**Table 2:** Bioactive constituents of ashwagandha and their pharmacological actions GABA: gamma-aminobutyric acid

Compound Name	Class	Pharmacological Role	Reference
Withaferin A	Withanolide	Anti-inflammatory (NF-κB inhibition), anticancer, and antioxidant	[10]
Withanolide A	Withanolide	Neuroprotective, promotes neurite outgrowth, and memory-enhancing	[15]
Sitoindoside IX & X	Glycowithanolides	Adaptogenic, stress-reducing, immunomodulatory	[2]
Withanoside IV	Glycoside	Enhances synaptic plasticity, learning, and memory	[19]
Somniferine	Alkaloid	Sedative and anxiolytic via GABAergic activity	[13]
Anaferine	Alkaloid	Immunomodulatory, mild antispasmodic	[6]
Iron (from root)	Mineral	Supports hematological balance, counters fatigue	[15]
Flavonoids (various)	Polyphenols	Antioxidant, anti-inflammatory synergy with withanolides	[5]

Adaptogenic mechanisms

Adaptogens are natural products that help organisms resist stress and maintain physiological balance. Brekhman and Dardymov established the core scientific standards for adaptogens: they must increase resistance to diverse stressors, restore balance regardless of the direction of dysfunction, and remain safe and non-toxic [19]. Ashwagandha meets these criteria by simultaneously regulating stress responses, immune activity, and neuroendocrine function. It has shown results equal to or better than *Rhodiola rosea, Eleutherococcus senticosus*, and *Panax ginseng* in lowering cortisol, enhancing stress resilience, and modulating immune markers [13,15]. Unlike other adaptogens, ashwagandha also promotes nerve repair and immune stability, thereby linking central and peripheral stress responses. A key mechanism involves modulation of the HPA axis, which governs the body’s stress response. Experimental studies in animal models demonstrated that ashwagandha extracts reduced stress-induced corticosterone surges, limited adrenal hypertrophy, and normalized dopamine, serotonin, and GABA levels, while preliminary human studies suggest similar neuroendocrine modulation [17]. Withanolides such as withaferin A appear to underlie these effects by suppressing corticotropin-releasing hormone (CRH) production and enhancing glucocorticoid sensitivity, thereby restoring HPA feedback loops [18,20]. Preclinical and clinical evidence support ashwagandha as a reliable adaptogen, particularly for conditions involving neuroendocrine and immune dysfunction.

Immunomodulation

Ashwagandha activates both the innate and adaptive arms of the immune system, enhancing overall immune surveillance and defense. Extracts of *Withania somnifera* stimulate natural killer (NK) cells, macrophages, and T lymphocytes [21]. Withanolides and sitoindosides drive these effects by promoting phagocytosis, expanding lymphocyte counts, and activating cytotoxic T cells in animal and human models [22]. At the molecular level, ashwagandha shifts the cytokine environment by increasing IL-2, IFN-γ, and TNF-α, while downregulating IL-6, IL-1β, and NF-κB [23,24]. These regulatory actions suggest that the herb balances immune activity by enhancing protective responses while limiting chronic inflammation. Preclinical and clinical data support these findings. In mice, ashwagandha root extract increased antibody production, delayed-type hypersensitivity, and macrophage-derived nitric oxide, confirming engagement of both innate and adaptive immunity [25]. In a randomized study of healthy adults (n=60), participants receiving 600 mg/day of KSM-66 extract for 60 days demonstrated significantly elevated NK cell activity and higher serum IgA and IgG compared to placebo [26]. Importantly, these effects occurred without pro-inflammatory reactions, underscoring the herb’s safety profile. Preliminary work also indicates potential benefits in autoimmune conditions. In animal models of rheumatoid arthritis and systemic lupus erythematosus, ashwagandha reduced disease activity by suppressing autoreactive T cells and inflammatory cytokines [27]. However, clinical evidence in autoimmune populations remains limited, warranting cautious interpretation until larger human studies are available. Taken together, these results highlight ashwagandha’s capacity to strengthen immunity while mitigating harmful inflammation, positioning it as a promising adjunct for stress-related and immune-mediated disorders. Table 3 summarizes key studies of its immunomodulatory activity in both preclinical and human models.

**Table 3:** Immunomodulatory activities of ashwagandha across experimental models FN-γ: interferon-gamma; IL: interleukin; TNF-α: tumor necrosis factor-alpha; IL-1β: interleukin-1 beta; NO: nitric oxide; IgA: immunoglobulin A; IgG: immunoglobulin G; PBMCs: peripheral blood mononuclear cells; NK: natural killer; LPS: lipopolysaccharide; IL-17A: interleukin-17A

Model Type	Immune Target	Observed Effect	Study Type	Reference
Murine splenocytes	T-helper (Th1/Th2) cytokines	Increased IFN-γ, IL-2; decreased IL-4, IL-10	In vivo (mice)	[2]
Human PBMCs	Natural killer (NK) cells	Enhanced NK cell cytotoxicity	Ex vivo	[26]
Equine model	TNF-α, IL-1β levels	Downregulated pro-inflammatory cytokines	In vivo (horses)	[21]
BALB/c mice	Macrophage activation	Increased phagocytosis and nitric oxide production	In vivo	[14]
Healthy humans	IgA, IgG serum levels	Elevated total immunoglobulin levels post-intervention	Clinical trial	[20]
RAW 264.7 cell line	IL-6, NO production	Suppressed inflammatory mediators under LPS challenge	In vitro	[11]
Autoimmune arthritis model	TNF-α, IL-17A	Reduced inflammatory cytokines; decreased joint inflammation	In vivo (rat)	[23]

Neuroprotection and cognition

**Figure 3:**
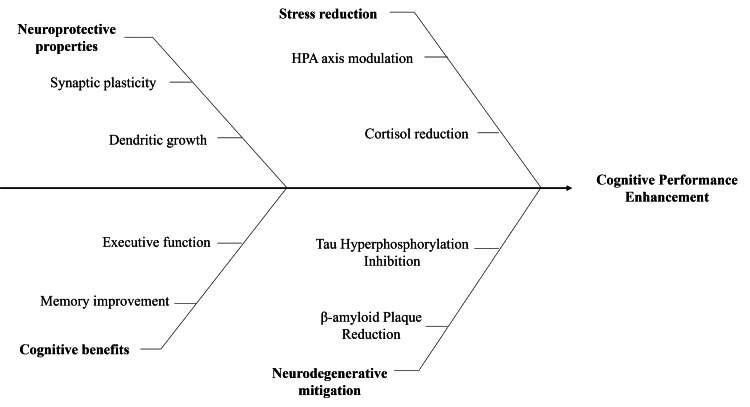
Mechanistic domains contributing to ashwagandha-mediated cognitive performance enhancement Created by authors using Microsoft PowerPoint (Microsoft Corporation, Redmond, Washington, United States) HPA: hypothalamic-pituitary-adrenal

Ashwagandha has been extensively studied for its neuroprotective effects and its ability to improve cognitive performance, particularly under conditions of stress and neurodegeneration. The herb’s bioactive constituents, especially withanolide A and withanoside IV, promote synaptic plasticity, dendritic growth, and axonal regeneration through antioxidant and anti-apoptotic pathways [28]. In rodent models, ashwagandha reversed scopolamine-induced memory loss and preserved hippocampal integrity, confirming its role in protecting cognition during oxidative stress [29]. Human trials have begun to mirror these findings. In a double-blind, placebo-controlled study (n=50), individuals with mild cognitive impairment who received 300 mg of root extract twice daily for eight weeks showed significant gains in immediate and general memory, executive function, and attention (p < 0.01), along with reduced serum cortisol levels [30]. The mechanisms underlying these effects likely involve regulation of the HPA axis, GABA mimetic activity, and anti-inflammatory cytokine modulation [28]. Preclinical evidence also suggests disease-modifying potential: ashwagandha reduced β-amyloid plaque accumulation, inhibited tau hyperphosphorylation, and controlled microglial overactivation, effects that may slow Alzheimer’s pathology [28,29]. Despite these encouraging results, human studies remain limited by small sample sizes, short durations, and variability in extract standardization and cognitive assessment methods. Larger, long-term randomized trials are needed to validate its therapeutic role. Ashwagandha demonstrates neuroprotective and cognitive benefits across both preclinical and early clinical studies, though stronger evidence is required for definitive clinical application. Figure 3 illustrates the multiple mechanisms through which ashwagandha enhances brain function, including synaptic plasticity, stress regulation, memory improvement, and protection against neurodegenerative processes.

Anxiolytic effects

Ashwagandha is increasingly recognized as a promising agent for anxiety and depression, acting through modulation of GABA signaling, regulation of the HPA axis, and reduction of inflammation. Withanolides, particularly withanolide A and withaferin A, interact with GABA_A receptors to enhance GABAergic transmission, producing anxiolytic effects similar to benzodiazepines but without sedation [31,32]. Clinical trials provide supportive evidence. In one study of 64 adults with chronic stress, participants receiving 300 mg of high-concentration ashwagandha root extract daily for 60 days experienced a 27.9% reduction in cortisol levels and significant improvements in stress scores [33]. Trials in patients with generalized anxiety disorder and subclinical depression also showed that ashwagandha improved Hamilton Anxiety Rating Scale (HAM-A) and Hamilton Depression Rating Scale (HAM-D) scores [34]. Furthermore, in individuals with moderate depression, combining selective serotonin reuptake inhibitors (SSRIs) with ashwagandha (500 mg/day) improved fatigue and cognitive function more than SSRIs alone [35]. Mechanistically, these effects are thought to arise from normalization of cortisol, enhancement of brain-derived neurotrophic factor (BDNF), and modulation of cytokines, all of which are dysregulated in affective disorders. Ashwagandha demonstrates anxiolytic and antidepressant potential across preclinical and clinical studies, though most trials remain limited by small sample sizes, short durations (four to 12 weeks), and heterogeneous extract preparations. While well-tolerated and promising, ashwagandha has yet to be confirmed for routine use in standard psychiatric care.

Ashwagandha and neurodegenerative disorders

**Figure 4:**
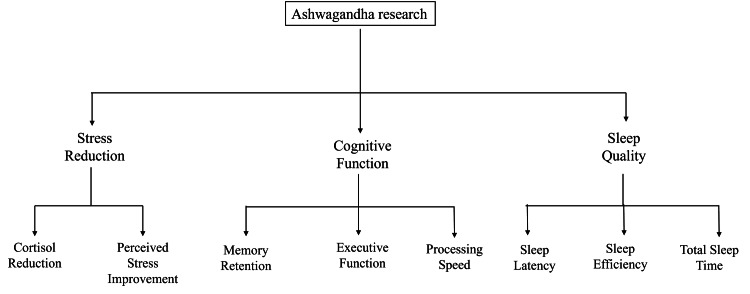
Clinical domains of ashwagandha’s therapeutic activity based on emerging research Created by authors using Microsoft PowerPoint (Microsoft Corporation, Redmond, Washington, United States)

Ashwagandha is under active investigation for its neuroprotective role in Alzheimer’s disease (AD), Parkinson’s disease (PD), and other neurodegenerative disorders. Its primary mechanisms include reducing oxidative stress, suppressing neuroinflammation, and limiting the accumulation of pathogenic proteins. Withanolide A and withanoside IV enhance antioxidant defenses by upregulating superoxide dismutase and glutathione peroxidase, thereby mitigating reactive oxygen species-mediated neuronal injury [36]. In preclinical AD models, ashwagandha reduced β-amyloid deposition, inhibited tau hyperphosphorylation, and restored synaptic connectivity [37]. Withanolide A further promoted neuronal growth and synaptic integration, improving cognitive performance in amyloid-induced Alzheimer’s mice [38]. In PD models, ashwagandha protected dopaminergic neurons by reducing α-synuclein aggregation, stabilizing mitochondrial function, and attenuating microglial activation and pro-inflammatory cytokine release (TNF-α, IL-1β) [39,40]. Despite these encouraging findings, significant translational barriers remain. Most supporting evidence arises from rodent and in vitro studies, which are limited by species-specific pharmacokinetics, variable extract composition, and poor bioavailability. As a result, direct clinical application in humans remains challenging. Current research in mild cognitive impairment and age-related memory decline shows early promise, but few studies have tested ashwagandha in patients with established AD or PD. Larger, long-term clinical trials are needed to determine efficacy, establish standardized dosing, and evaluate long-term safety in neurodegenerative populations. Ashwagandha exerts multi-target neuroprotective effects that make it a compelling candidate for adjunctive therapy in neurodegenerative diseases. However, its integration into clinical practice depends on overcoming methodological and translational limitations through rigorous human studies. Figure 4 illustrates the herb’s therapeutic domains across stress modulation, cognition, and sleep, supported by biomarker and behavioral evidence from recent trials.

Sleep and circadian rhythm

In Ayurvedic medicine, ashwagandha is classified as a “*nidrajanana rasayana*,” a rejuvenative formulation traditionally prescribed to restore nervous system homeostasis and alleviate stress-related insomnia [41]. Contemporary pharmacological studies corroborate this designation, demonstrating that ashwagandha modulates key neurotransmitter systems, including GABAergic and serotonergic pathways, that regulate sleep architecture. Preclinical experiments in animal models further reveal that ashwagandha administration significantly prolongs non-rapid eye movement (NREM) sleep and reduces sleep latency, thereby providing mechanistic support for its sleep-promoting effects [42]. These effects are attributed to interactions at GABA A and 5-HT 1A receptors, which are also primary targets of benzodiazepines, though ashwagandha appears to achieve sedation without the same motor side effects. In particular, a triethylene glycol (TEG) fraction of ashwagandha induced sleep in animal models without impairing motor coordination, distinguishing it from conventional hypnotics [43]. Emerging evidence suggests that ashwagandha may also regulate circadian rhythms. In vitro studies on suprachiasmatic nucleus (SCN) neurons showed that ashwagandha extract upregulated clock genes BMAL1 and PER2, indicating potential support for biological rhythm stability under stress [44]. Human trials have extended these findings: in a randomized study of 150 adults with insomnia, participants who received 600 mg/day of ashwagandha extract for eight weeks reported significant improvements in sleep efficiency, total sleep time, and sleep onset latency compared with placebo (p < 0.01) [45]. They also reported better daytime alertness with minimal residual drowsiness [46]. Despite these promising short-term outcomes, long-term effects remain poorly understood. Few studies have examined whether improvements persist after discontinuation or whether circadian gene regulation translates into clinical benefits in patients with comorbid conditions [47]. Ashwagandha demonstrates sleep-promoting and circadian-modulating properties with evidence from both preclinical and clinical studies, but further long-term trials are necessary to confirm the durability and safety of these effects.

Anti-inflammatory pathways

**Figure 5:**
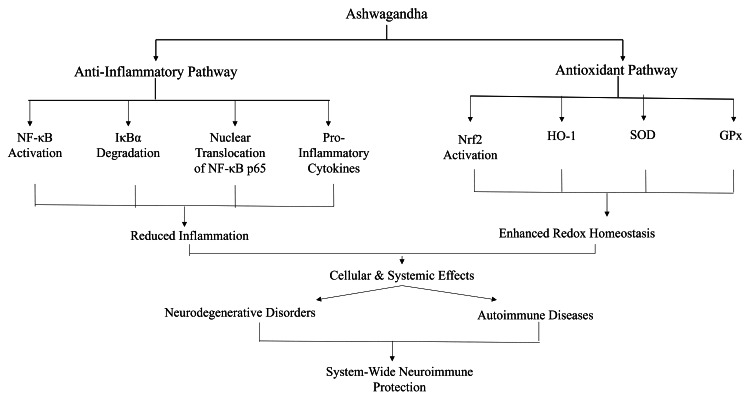
Dual anti-inflammatory and antioxidant pathways of ashwagandha leading to neuroimmune protection Image credit: Pallavi Prabhakar Jamnekar

Ashwagandha exhibits both anti-inflammatory and antioxidant properties, making it a promising intervention for disorders driven by oxidative and inflammatory stress [48]. These effects are primarily mediated through modulation of NF-κB and Nrf2 signaling pathways, which are critical for immune balance and redox homeostasis [49]. Withaferin A, a major bioactive constituent, inhibits NF-κB activation by preventing IκBα degradation and blocking NF-κB p65 translocation to the nucleus. This leads to reduced expression of pro-inflammatory mediators such as IL-1β, TNF-α, and COX-2 [50]. Concurrently, ashwagandha activates the Nrf2-ARE pathway, which upregulates antioxidant enzymes including HO-1, SOD, and GPx [51]. Experimental data reinforce these mechanisms. In vitro studies with neuronal and immune cells show that ashwagandha lowers intracellular reactive oxygen species (ROS), stabilizes mitochondrial membranes, and enhances cell survival under oxidative stress [22]. Animal studies further demonstrate protection against glial activation, blood-brain barrier disruption, and neuroinflammation in lipopolysaccharide (LPS)-induced neurotoxicity models [5]. These actions are particularly relevant where the nervous and immune systems intersect, such as in neurodegenerative and autoimmune disorders. Ashwagandha’s ability to simultaneously regulate transcription factors and inflammatory mediators underscores its dual benefit for both immune and neural cells [14]. Because cytokines, ROS, and NF-κB serve as shared mediators between these systems, such regulation is crucial in conditions involving immune-neural dysfunction [35]. Despite robust preclinical evidence, clinical validation is limited. Large-scale human trials are still needed to confirm efficacy, optimize dosing, and translate these findings into therapeutic strategies for inflammatory and oxidative stress-related disorders. Ashwagandha modulates NF-κB and Nrf2 signaling to reduce inflammation and strengthen antioxidant defenses. Figure 5 illustrates these dual mechanisms, highlighting their relevance to neuroimmune protection and potential applications in chronic inflammatory and neurodegenerative diseases.

Clinical trials

Randomized controlled trials (RCTs) provide growing evidence for ashwagandha’s benefits in stress reduction, cognitive enhancement, sleep quality, and overall well-being. Across trials, results are generally supportive, though study designs vary in dosage, formulation, duration, and participant demographics. One RCT enrolled 64 chronically stressed adults who received 600 mg/day of KSM-66 root extract for 60 days, resulting in a 27.9% decrease in serum cortisol and significant reductions in stress scores compared with placebo [33]. In patients with generalized anxiety disorder, ashwagandha treatment for six weeks reduced HAM-A scores by 56% of lorazepam’s effect, but without sedation [34]. Sleep-related benefits were demonstrated in a study of 150 adults with insomnia, where 600 mg/day of root extract for eight weeks significantly improved sleep onset, total sleep time, and sleep efficiency (p < 0.01) [45]. Cognitive benefits have also been reported: in individuals with mild cognitive impairment (MCI), 300-500 mg twice daily for eight to 12 weeks enhanced memory, executive function, and processing speed [30].

Formulation and dosing patterns vary across trials. Most clinical studies use KSM-66 (aqueous root extract) or Sensoril (root-leaf blend with higher withanolide content) [17,18]. KSM-66 is primarily studied for stress and endurance outcomes, while Sensoril has been tested for metabolic and cognitive endpoints. Dosing regimens typically range from 250 to 1,000 mg/day for four to 12 weeks. Study populations include healthy adults under stress, older adults with memory impairment, and clinical cohorts with insomnia, schizophrenia, hypothyroidism, or infertility [26,35]. Most participants are 25-65 years old, with balanced gender representation, and are primarily from South Asia, North America, and the Middle East.

A systematic review analyzed multiple RCTs and concluded that ashwagandha has a moderate positive impact on stress reduction and cognitive performance. However, variability in study design, outcome measures, and extract formulations limited the strength of the overall conclusions [23]. Table 4 consolidates key RCTs of ashwagandha, summarizing formulation, dose, duration, and therapeutic outcomes across neurological, immunological, and stress-related domains. RCTs consistently demonstrate ashwagandha’s clinical potential, but variability in methodology and formulations highlights the need for larger, standardized trials to confirm efficacy and support regulatory acceptance.

**Table 4:** Summary of key clinical trials on ashwagandha QoL: quality of life; SR: sustained release; GAD-7: generalized anxiety disorder-7; PSS: Perceived Stress Scale; HAM-A: Hamilton Anxiety Rating Scale; HRV: heart rate variability

Population Studied	Extract/Formulation	Dose & Duration	Key Outcomes	Reference
Healthy adults	Standardized root extract	600 mg/day, 60 days	Improved immune markers, IL-6 and IgG levels	[26]
Healthy adults	KSM-66 root extract	600 mg/day, 8 weeks	Reduced cortisol, improved anxiety, and QoL scores	[45]
Adults with mild anxiety	Ethanol root extract	250–600 mg/day, 6–8 weeks	Significant reduction in GAD-7 and PSS scales	[4]
Geriatric dogs (model)	Ashwagandha SR formulation	500 mg/day, 12 weeks	Improved cognition and reduced inflammation biomarkers	[36]
Adults with generalized anxiety	Aqueous root extract	300 mg BID, 60 days	Reduced HAM-A scores; improved sleep latency and HRV	[25]
Healthy volunteers	Rasayana root preparation	3–6 g/day, 30 days	General well-being, fatigue reduction	[11]
Adults with chronic stress	KSM-66 ashwagandha root extract	600 mg/day (300 mg twice daily) for 60 days	27.9 % reduction in serum cortisol; significant reductions in stress scores vs. placebo	[33]
Stressed adult population	Withania capsule	600 mg/day, 4 weeks	Better sleep quality, improved cortisol rhythm	[20]
Adults with chronic stress	Commercial supplement blend	Variable dose, 4 weeks	Reduction in fatigue and salivary cortisol	[16]

Safety

Ashwagandha is generally safe and well-tolerated, with evidence from both preclinical and clinical studies. In rodent experiments, oral LD₅₀ values exceeded 2,000 mg/kg, and no behavioral or systemic toxicity was observed at high doses [11,12]. Subchronic studies lasting more than 90 days also showed no adverse effects on the liver, kidneys, or hematological parameters [16]. Clinical trials report mostly mild and infrequent adverse events (AEs). Across studies, only 5-8% of participants experienced AEs, typically gastrointestinal upset, drowsiness, or headache [12]. No clinically significant changes in hepatic, renal, or hematological function have been observed with up to 12 weeks of supplementation [12]. Nevertheless, herb-drug interactions warrant caution. Due to its GABAergic activity, ashwagandha may potentiate the sedative effects of CNS depressants or benzodiazepines [32]. In individuals with subclinical hypothyroidism, supplementation significantly reduced thyroid-stimulating hormone (TSH) levels, suggesting possible interactions with thyroid medications [26]. Because ashwagandha enhances immune activity by increasing NK cell function and cytokine levels [21], it may be unsuitable for transplant patients or those receiving immunosuppressants.

Special populations also require caution. Animal studies indicate that high doses can stimulate the uterus and cause abortion, making ashwagandha unsafe during pregnancy [13]. Insufficient data exist for children, and use should be avoided without medical supervision. In older adults, ashwagandha has shown benefits for fatigue, cognition, and sleep, though absorption and metabolism may vary with age [28,30]. Finally, standardization of extracts is essential for evaluating safety. Differences in withanolide concentration, plant part used (root vs. leaf), and extraction methods affect both bioactivity and tolerability [17,18]. Thus, findings from one preparation cannot be generalized to all formulations. Ashwagandha is safe when taken as directed in controlled studies, but long-term safety and interactions in polypharmacy populations remain areas for further research.

Limitations

Significant heterogeneity across studies posed challenges in data interpretation. The studies used different dosages (from 250 mg to 1,000 mg/day) and extracts (KSM-66 root-only and Sensoril root-leaf) and measured various outcomes (cortisol, HAM-A, sleep latency, and memory recall). For example, KSM-66 was given at 600 mg/day in studies on stress, while Sensoril was given at 250 mg/day in studies on metabolism and thinking. Because the studies are not all the same, it is hard to compare them and use the results widely. Also, many important results from animal studies, especially those related to protecting the brain or immune system, may not be directly applicable to people because of differences in metabolism and immune system complexity. A further concern involves the risk of bias. Many studies with positive results were sponsored by companies that produce ashwagandha extracts, which could lead to some problems with reporting and not including all adverse effects. Negative or null studies are less likely to be published in journals, which can lead to a biased view of the literature. This means that more clinical trials should be done in multiple centers, with bigger groups of patients, standardized extracts, and strict trial rules.

Future directions

Researchers should focus on explaining ashwagandha’s actions on the immune and nervous systems by studying its molecular pathways with advanced methods like transcriptomics, proteomics, and Clustered Regularly Interspaced Short Palindromic Repeats (CRISPR)-based pathway mapping. In addition, personalized approaches should be studied, especially to find out how variations in genes like NR3C1 and GABRA1 can influence the effectiveness of treatments, requiring the use of pharmacogenomic data. A major issue is that standardization is still lacking; differences in withanolide content, how the extracts are made, and the plant parts used make it hard to repeat studies. Future studies should use good manufacturing practice (GMP)-grade, chemically defined extracts to deal with these issues. To validate how effective a drug is, it is important to conduct long-term, multicenter randomized controlled trials with different populations and use objective measures, track biomarkers, and ensure safety. All of these studies will help ashwagandha move forward from being used in traditional ways to being used as a precise, scientifically supported treatment and will support its consideration for regulation and clinical application.

## Conclusions

Ashwagandha has well-documented potential as an adaptogenic plant with clinically significant activity on immune function and neurological health. Its rich phytochemical content, comprising withanolides, sitoindosides, and alkaloids, forms the basis for its activity in numerous physiological systems, such as the HPA axis, cytokine modulation, GABAergic pathways, and regulation of oxidative stress. Translational research invariably documents improved immune surveillance, decreased pro-inflammatory markers, and enhanced neurogenesis. These results are supported by pilot clinical trials with up to 28% reductions in serum cortisol, improved sleep quality, and quantifiable gains in cognitive function within eight to 12 weeks of treatment. Combined, this evidence supports ashwagandha's utility in treating conditions underpinned by neuroinflammation, immune dysregulation, and stress-associated neural dysfunction.

However, significant limitations persist. Extract standardization variability, outcome measures, and study duration continue to influence data consistency and generalizability. Finally, extrapolating from animal models to human pathophysiology must be approached with caution owing to differences in immune and metabolic responses between species. Long-term, multicenter randomized controlled trials with standardized formulations, representative participant cohorts, and combined pharmacodynamic analyses and biomarker follow-up should be the focus of future research to elucidate underlying mechanisms. Ashwagandha's therapeutic potential can be optimized within tailored, evidence-based care models that integrate botanical therapeutics with genomic testing and lifestyle intervention. With ongoing rigorous investigation and cross-disciplinary collaboration, ashwagandha may emerge as a validated botanical therapy, complementing conventional approaches within integrative, evidence-based medicine.
